# The War and Sexual Morality

**Published:** 1914-11

**Authors:** 


### The War and Sexual Morality.

It is reported that the Germans have 1,700,000 men now in training to be thrown into the active fighting before the first of December, and that immediately thereafter the boys of the Empire, to the number of more than half a million, will be put into the ranks for drill, that they may enter active service in the spring.

The death rate is appalling and unprecedented, with the stubbornness of the fray, combined with the deadly execution of modern equipment.

The future looks dark according to our conventional ideas of sexual morality.

Not alone the ordinary human impulse will subvert the normal standards, but economic conditions will demand the birth of children to fill the wide gaps in population, will wink at what would otherwise prove unanswerable arguments for relations otherwise inconceivable.

The worst of the war is not in its loss of life, its sundering of ties, its woeful destruction of art and property, its upheaval destruction of inventive and manufacturing skill, plants and business and of the accumulated wealth of centuries.

The worst will be felt for a century, in a lowered morality, even as the Napoleonic wars have left a record of illegitimacy and lax sexual morality which has marred France to the present day.

